# Effect of feed supplement and additives on stress mitigation in Karan Fries heifers

**DOI:** 10.14202/vetworld.2017.1407-1412

**Published:** 2017-12-01

**Authors:** Vaibhav Purwar, P. S. Oberoi, A. K. Dang

**Affiliations:** 1 Livestock Production and Management Division, ICAR-National Dairy Research Institute, Karnal - 132 001, Haryana, India; 2 Animal Physiology Division, ICAR-National Dairy Research Institute, Karnal - 132 001, Haryana, India

**Keywords:** feed supplement, heat stress, hot humid, Karan Fries

## Abstract

**Aim:**

The objective of this study was to evaluate the effects of protected fat plus yeast, niacin, zinc, and chromium dietary supplementation on the reduction of heat stress in Karan Fries (KF) heifers during hot humid months.

**Materials and Methods:**

The basal ration for both the control and treatment groups was the same, containing maize as green fodder and concentrate mixture. However, the treatment group was supplemented with protected fat (2.5% of dry matter intake [DMI]), yeast (10 g/animal/day), niacin (6 g/animal/day), zinc (40 mg/kg DMI), and chromium (1.5 mg/kg DMI).

**Result:**

The overall mean value of afternoon rectal temperature for control and treatment group was 103.17±0.09 and 102.72±0.10°F, respectively, and was significantly (p<0.01) lower in the treatment group. The overall mean value of afternoon respiration rate for control and treatment group was 76.35±0.56 and 73.13±0.58 breaths/min, respectively, and was also significantly (p<0.01) lower in the treatment group. The overall mean value of afternoon pulse rate for control and treatment group was 97.09±0.63 and 94.67±0.67 beats/minute, respectively, and was also significantly (p<0.01) lower in the treatment group. Finally, the mean cortisol concentration for control and treatment group was 3.94±0.05 ng/ml and 3.70±0.06 ng/ml, respectively, and was significantly (p<0.01) lower in the treatment group.

**Conclusion:**

The present study shows that supplementation with the above feed additives could serve as a heat stress abatement strategy in growing KF heifers during extreme conditions in summer months.

## Introduction

The mean temperature of the earth is continuously rising due to the effect of global warming. In a study conducted by IPCC [1], the earth temperature has been elevated by 0.85°C (0.65-1.06) during the period from 1880 to 2012. It is also predicted that the earth temperature will be further increased due to the continuous emission of greenhouse gases. The climatic parameters will be also changed with further increase of temperature, which will impact the growth and production of domestic livestock. Due to global warming, management of the heat stress in domestic animals has become an important issue not only for the tropical nations but also for temperate countries. Kibler and Brody [2] studied the energy requirement under heat stress conditions and observed that maintenance energy requirements of animal were enhanced by 7% during heat stress. Heat tolerance of crossbred cattle (*Bos taurus* crosses) is poorer as compared to indigenous cattle (*Bos* *indicus*) because of lesser sweat gland density and lesser surface area per unit weight.

Heat stress causes an imbalance between reactive oxygen species (ROS) and antioxidants, which leads to oxidative stress. ROS induces damage of cells which further causes a decline in animal performance, especially during heat stress conditions [3]. Zinc addition improves the antioxidant status and thus ameliorates the oxidative stress [4]. Stress also increases cortisol level, and it has been observed that chromium addition in the diet of stressed cattle decreases serum cortisol levels [5]. During stress, chromium excretion increases in urine [6], and hence, its exogenous addition becomes necessary. Liu *et al*. [7] also reported that the detrimental effect of heat stress can be reduced by chromium supplementation in pigs. Chromium supplementation increases the insulin activity and improves glucose utilization [8].

The effect of fat supplementation on physiological parameters of animal is variable since some researchers reported a decrease in rectal temperature (RT) and respiration rate (RR) [9] while others reported no effect [10]. Niacin plays a significant role in abatement of heat stress by increasing peripheral circulation and sweating [11,12]. Ducray *et al*. [13] showed a protective effect of the yeast fermentate product in rats for prevention of complications, caused by heat stress. Das *et al*. [14] also demonstrated that dietary supplementation with yeast, niacin, and mustard oil along with some other management practices resulted in alleviating summer stress in Nili Ravi buffaloes.

The major strategies for reducing heat stress such as high insulated roof housing, sprinklers, fans, and air conditioner require a high financial investment and may not be applicable for small and medium-size dairy farms. Thus, there is a need of applying other management practices to reduce thermal stress, such as incorporation of feed additives that could improve productivity of the animals. Information regarding the individual use of different supplements and additives on summer stress is available; however, the concomitant use of these supplements has not yet been studied in crossbred cattle.

As a result, the present study was designed to evaluate the potential of a mix of several feed additives in reducing heat stress in Karan Fries (KF) heifers during hot humid months.

## Materials and Methods

### Ethical approval

The experiment was carried out according to the National Regulations on Animal Welfare and the Institutional Animal Ethical Committee.

### Study design

The study was carried out at Livestock Research Center, National Dairy Research Institute, Karnal for 90 days on 18 KF heifers. The heifers were selected randomly and distributed into treatment and control groups based on the body weight. Each group comprised of nine animals. At the beginning of the experiment, the average age and body weight of the heifers in treatment group were 372.44±18.07 days and 125.32±7.46 kg and in control group were 377.33±13.11 days and 125.55±8.27 kg, respectively. The animals were clinically healthy and kept under the same conditions, with appropriate facilities for feeding and watering. The basal ration for both the control and treatment group was the same, containing maize as green fodder and concentrate mixture. The treatment group was also supplemented with protected fat (2.5% of dry matter intake [DMI]) [15], yeast (10 g/animal/day) [14], niacin (6 g/animal/day) [14], zinc (40 mg/kg DMI) [16], and chromium (1.5 mg/kg DMI) [17]. Total mixed ration (TMR) was prepared and fed in two equal meals in the morning and in the evening. The nutrient requirement suggested by ICAR [16] was considered for ration formulation. The required amount of feed additives was added in the TMR prepared from green fodder and concentrated mixture. The effect of weather parameters, such as temperature and humidity, on the KF heifers was expressed in terms of temperature humidity index (THI). Observations for THI were recorded at 8:30 am and 4:22 pm daily. The average THI was weekly calculated for the experimental period using the following formula [18]:

THI=0.72 (db°C+wb°C)+40.6

Where db°C - dry bulb temperature (°C), and wb°C - wet bulb temperature (°C).

The RT, RR, and pulse rate (PR) were weekly measured in the morning and in the evening. The RTs (°F) of both the groups were recorded using a digital thermometer by inserting 3 inches in the rectum for about 2 min. The RR per minute was recorded by counting the movement of flank, i.e., one outward and inward movement considered as one respiration. The PR per minute was counted by palpating coccygeal artery. The normal values for RTs, RR, PR, and THI are summarized in Table-1 [19-21]. Blood samples were collected fortnightly, and plasma was separated and stored at −20°C for estimation of cortisol levels using commercially available kits.

**Table-1:** Normal range of parameters.

Parameters	Normal range
Rectal temperature [19]	100.4-102.8°F
Respiration rate [19]	24-50 cycles/min
Pulse rate [20]	60-80 beats/min
THI [21]	<72

THI=Temperature humidity index

### Statistical analysis

The statistical analysis was performed using T-test in SPSS software (16.0). Bivariate Pearson correlation analysis was also employed.

## Results

The values for THI are summarized in Table-2. The values of morning THI observations varied from 60.60±0.66 to 79.79±0.50, and afternoon observations values varied from 74.81±0.53 to 84.60±0.65 during the present study. The mean value of RT is presented in Table-3. The overall mean value of RT in the morning was 101.47±0.06°F for control and 101.31±0.07°F for supplemented animals (no significant difference (p>0.05) was observed). The overall mean value of RT in the afternoon was 103.17±0.09 and 102.72±0.10°F for the control and the treatment group, respectively, and was significantly (p<0.01) different. The mean values of RR are presented in Table-4. The overall mean value of RR in the morning showed no significant (p>0.05) difference (46.95±0.56 vs. 45.55±0.61 breaths/min for control and treatment group, respectively). The respective values in the afternoon were 76.35±0.56 and 73.13±0.58 breaths/min in control and treatment group animals and were significantly (p<0.01) different. The mean values of PR are presented in Table-5. The overall mean value of PR in the morning was 77.56±0.66 and 75.85±0.62 beats/min for the control and the supplemented group, respectively, and no significant difference (p>0.05) was observed. The overall mean values of PR in the afternoon were significantly different (97.09±0.63 vs. 94.67±0.67 beats/min for control and treatment group, respectively) animals. The correlation analysis between a physical parameter of stress and THI is summarized in Table-6. The mean value of cortisol is presented in Table-7. The overall mean of fortnightly blood plasma cortisol concentrations was recorded as 3.94±0.05 and 3.70±0.06 ng/ml for control and supplemented animals, respectively, and was significantly different (p<0.01).

**Table-2:** THI values for the morning (AM) and afternoon (PM) during experimental period.

Week	AM	PM
1	79.79±0.50	84.34±0.83
2	78.39±0.13	81.66±0.82
3	79.52±0.75	82.68±1.01
4	78.20±0.32	80.88±1.17
5	79.15±0.36	84.01±0.31
6	78.29±0.73	82.09±1.07
7	76.58±0.39	83.04±0.30
8	76.46±0.35	83.49±0.24
9	78.35±0.38	84.60±0.65
10	76.48±0.36	82.50±0.66
11	77.83±0.57	84.27±0.32
12	72.36±1.08	81.48±0.61
13	66.64±0.48	79.13±1.23
14	66.54±0.32	78.43±0.33
15	63.48±0.46	75.98±0.27
16	60.60±0.66	74.81±0.53
17	58.54±1.03	73.56±0.44

THI=Temperature humidity index

**Table-3:** Weekly morning (AM) and afternoon (PM) rectal temperature (°F) in KF heifers.

Week	Control (AM)	Treatment (AM)	Control (PM)	Treatment (PM)
0	102.02±0.26	102.08±0.21	104.58±0.22	104.27±0.20
1	102.14±0.19	102.11±0.20	103.22±0.17	102.96±0.17
2	101.59±0.14	101.47±0.24	103.79±0.23	103.03±0.38
3	101.54±0.12	101.34±0.26	103.30±0.22	102.91±0.12
4	101.71±0.24	101.59±0.26	103.10±0.12	102.64±0.19
5	101.36±0.18	101.22±0.29	103.79±0.29	103.29±0.22
6	101.71±0.20	101.50±0.24	103.15±0.14	102.74±0.17
7	101.33±0.26	100.97±0.23	104.09±0.29	103.52±0.25
8	101.5±0.21	101.37±0.19	103.28±0.25	102.78±0.29
9	101.57±0.19	101.42±0.18	103.52±0.23	103.02±0.28
10	101.18±0.22	100.92±0.21	102.48±0.14	102.19±0.26
11	101.39±0.20	101.10±0.24	102.34±0.16	101.86±0.24
12	100.84±0.17	100.69±0.18	102.08±0.16	101.67±0.29
13	100.69±0.20	100.53±0.16	101.68±0.14	101.17±0.24
Mean±SE	101.47±0.06	101.31±0.07	103.17±0.09^a^	102.72±0.10^b^

Values with different superscript differ significantly (p<0.01). SE=Standard error, KF=Karan Fries

**Table-4:** Weekly morning (AM) and afternoon (PM) respiration rate (breaths/min) in KF Heifers.

Week	Control (AM)	Treatment (AM)	Control (PM)	Treatment (PM)
0	53.22±1.65	51.56±1.74	84.67±1.32	83.89±1.29
1	48.67±1.45	47.44±1.54	75.78±1.79	73.56±1.20
2	50.56±1.63	50.22±2.13	79.67±1.86	76.00±1.67
3	49.78±1.65	47.78±1.82	73.44±1.78	70.56±1.94
4	49.00±1.35	46.33±1.76	74.67±2.02	72.11±1.35
5	49.56±1.40	50.89±1.96	78.56±1.29	74.00±2.11
6	46.11±1.07	44.56±1.53	72.44±2.01	68.33±1.98
7	44.44±2.04	42.44±1.48	79.00±1.72	76.22±1.96
8	47.22±1.16	44.56±1.72	78.00±1.55	73.78±1.53
9	50.56±1.63	50.67±1.91	79.78±1.89	74.78±1.54
10	48.78±1.50	47.44±1.54	76.33±2.03	72.11±1.64
11	42.89±1.90	40.22±2.00	78.89±1.20	77.11±1.06
12	40.33±1.88	38.56±1.32	70.67±1.72	66.11±1.50
13	36.22±1.16	35.11±1.43	68.78±1.79	65.22±0.97
Mean±SE	46.95±0.56	45.55±0.61	76.35±0.56^a^	73.13±0.58^b^

Values with different superscript differ significantly (p<0.01). SE=Standard error, KF=Karan Fries

**Table-5:** Weekly morning (AM) and afternoon (PM) pulse rate (beats/minute) in KF Heifers.

Week	Control (AM)	Treatment (AM)	Control (PM)	Treatment (PM)
0	82.56±1.45	82.67±1.44	102.78±1.86	101.11±2.26
1	86.33±1.91	85.11±2.09	97.67±2.19	94.56±1.40
2	79.89±3.33	78.67.±2.81	99.56±1.83	97.67±1.91
3	76.89±2.10	74.78±1.64	97.56±2.43	95.56±2.45
4	81.56±2.20	79.67±1.65	96.00±2.13	93.22±2.33
5	77.33±1.91	74.33±2.01	98.67±1.89	96.44±1.79
6	75.22±1.47	73.44±1.55	90.56±2.14	88.00±1.76
7	78.00±3.50	76.89±1.84	98.00±1.91	95.78±2.03
8	77.11±1.86	75.22±1.90	99.11±2.93	96.67±3.36
9	78.67±2.73	78.00±1.73	100.78±2.22	99.78±2.56
10	75.22±1.47	72.56±1.62	97.22±2.13	93.89±2.02
11	75.78±1.77	72.78±1.50	97.56±1.61	96.33±2.07
12	72.78±1.39	70.22±1.81	93.00±2.67	89.67±2.43
13	68.44±1.26	67.56±1.61	90.78±2.53	86.78±2.34
Mean±SE	77.56±0.66	75.85±0.62	97.09±0.63^a^	94.67±0.67^b^

Values with different superscript differ significantly (p<0.01). SE=Standard error, KF=Karan Fries

**Table-6:** Correlation coefficient between THI and physical parameters of stress.

Parameter		Control		Treatment	
	
		Value of r	Level of significance	Value of r	Level of significance
THI (AM)		RT (AM)	0.824	**	0.785	**
THI (PM)		RT (PM)	0.647	*	0.621	*
THI (AM)		RR (AM)	0.956	**	0.897	**
THI (PM)		RR (PM)	0.940	*	0.908	*
THI (AM)		PR (AM)	0.801	*	0.762	*
THI (PM)		PR (PM)	0.989	*	0.911	*

RT=Rectal temperature, RR=Respiration rate, PR=Pulse rate, THI=Temperature humidity index.

* p<0.05,

** p<0.01

**Table-7:** Fortnightly cortisol concentration (ng/ml) in KF Heifers.

Fortnight	Control	Treatment
0	4.19±0.20	4.06±0.18
1	4.13±0.16	3.94±0.15
2	4.06±0.19	3.79±0.22
3	3.93±0.13	3.67±0.11
4	3.81±0.10	3.55±0.12
5	3.77±0.11	3.48±0.10
6	3.69±0.12	3.43±0.14
Mean±SE	3.94±0.05^a^	3.70±0.06^b^

Values with different superscript differ significantly (p<0.01). SE=Standard error, KF=Karan Fries

## Discussion

The values of THI, RT, RR, and PR were beyond the normal range which indicates that the animals were in the state of heat stress [21]. The THI values were gradually decreased as the season changed from summer to autumn. The mean value of RT, RR, and PR was lower during morning hours due to the higher heat dissipation rates during night hours. The mean value of RT, RR, and PR differ significantly (p<0.01) in the afternoon between treatment and control animals. The observed difference in RT, RR, and PR was possibly an effect of additives and bypass fat supplementation. Suraj [15] found a significant (p<0.01) decrease in RT in 2.5% prilled fat supplemented KF heifers. Zimbelman *et al*. [22] also reported that supplementation with rumen-protected niacin (12 g/d) in dairy cows during summer resulted in a significant (p<0.01) decrease in vaginal temperature in supplemented (38.38°C) compared to control (38.53°C) group. According to Zhu *et al*. [23], RT tended to linearly decrease (p=0.07) in cows supplemented with *Saccharomyces cerevisiae* at 14:30 but not at 06:30 (p>0.10). Das *et al*. [14] offered additional niacin (6 g/buffalo/day), yeast (10 g/buffalo/day), and mustard oil (150 g/buffalo/day) during summer in Nili Ravi buffaloes and found a significant decrease in RT, RR, and PR. Broadway *et al*. [24] also found a decrease in vaginal temperature (p<0.01) and RR (p=0.09) in live yeast (1.5 g/animal/d) supplemented beef heifers, during summer months. Gyanendra *et al*. [25] showed a significant (p<0.05) reduction in RR, PR in buffalo calves supplemented with zinc sulfate (500 mg/animal/day). Singh *et al*. [26] supplemented yeast and found a reduction in PR in the supplemented group. Wrinkle *et al*. [27] also found a reduction in morning RR and afternoon PR (p≤0.01) as a result of rumen-protected niacin supplementation (19 g/day). Rejeb *et al*. [28] reported that, for each point increase in RT value above 38.5°C, there was a decrease in DMI of 1.31 kg/day. This decrease in DMI will negatively affect the performance of animal. The decrease in RT, RR, and PR in animals fed with feed additives/supplements indicates improved thermotolerance and better performance. The positive correlation values indicate that, when THI increases, RT, RR, and PR were also increased. The higher value of correlation coefficient in control group animals shows that the increase in RT, RR, and PR was greater in control than supplemented animals (level of significance was not different). The lower values for RT, RR, and PR in treatment group animals possibly suggest that there is the effect of supplementation on summer stress alleviation.

The mean cortisol concentration was reduced as the experiment continued in both treatment and control group due to the decrease of THI. However, the mean cortisol concentration differs significantly (p<0.01) between the control and treatment groups. The decrease in cortisol concentration in treatment group was possibly an effect of the use of additives in the ration of treatment group. Kumar *et al*. [17] reported a decrease in cortisol blood concentration as a result of chromium supplementation. Kegley *et al*. [5] also demonstrated decreased serum cortisol level in cattle after the chromium addition. Pechova *et al*. [8] supplemented dairy cows with 0.5 ppm chelated chromium and observed a reduction in cortisol levels. Chang *et al*. [29] demonstrated a reduction in blood plasma cortisol level in calves supplemented with organic chromium. Kumar *et al*. [30] supplemented 24 buffalo calves for 120 days with different chromium concentrations (0, 0.5, 1.0, and 1.5 ppm) and found a decrease in blood plasma cortisol levels. Suraj [15] also found a significant (p<0.01) decrease in cortisol levels in KF heifers supplemented with 2.5% prilled fat. Patel [31] found a significant (p<0.05) decrease in cortisol content in pregnant KF cows supplemented with 80 and 120 ppm zinc. Finally, according to Broadway *et al*. [24], blood plasma cortisol levels in beef heifers during heat stress months tended to decrease (p=0.08) after the supplementation with live yeast (1.5 g/hd/d).

## Conclusion

The present study clearly indicates that, during the periods of summer stress, KF heifers show an elevation in their RT, RR, PR, and plasma cortisol levels. Supplementation with protected fat, yeast, niacin, zinc, and chromium enables them to reduce the values of these physiological parameters. Application of these supplements in a large number of cows will further help us to understand the complex relationships between the environmental variables and physiological parameters.

The results of present study suggest that the supplementation with protected fat (2.5% of daily DMI), yeast (10 g/animal/day), niacin (6 g/animal/day), zinc (40 mg/kg DMI), and chromium (1.5 mg/kg DMI) in the ration of growing heifers during summer stress period can ameliorate the effect of summer stress and minimize fiscal losses of the farmer.

## Authors’ Contributions

All authors contributed in the planning and doing research work as follows: VP (study design, doing, statistics, and writing), PSO (study design, doing, and writing), and AKD (study design, doing, writing). All authors read and approved the final manuscript.
